# Combining breastfeeding and work: findings from the Epifane population-based birth cohort

**DOI:** 10.1186/s12884-020-2801-x

**Published:** 2020-02-17

**Authors:** Katia Castetbon, Julie Boudet-Berquier, Benoit Salanave

**Affiliations:** 1 Université libre de Bruxelles, Ecole de Santé Publique, Centre de Recherche en Epidémiologie, Biostatistique et Recherche Clinique, CP 598, Université libre de Bruxelles, Route de Lennik, 808, B-1070 Bruxelles, Belgium; 2 Equipe de Surveillance et d’Epidémiologie Nutritionnelle (Esen), Santé Publique France, Université Paris 13, Centre de Recherche en Epidémiologie et Statistiques, COMUE Sorbonne Paris Cité, Bobigny, France; 3 Département des Maladies Non Transmissibles et Traumatismes (DMNTT), Santé Publique France, Saint Maurice, France

**Keywords:** Birth cohort, Breastfeeding, Employment, Maternity leave, Mothers, Social determinants

## Abstract

**Background:**

Return to work is often cited as a reason for early cessation of breastfeeding (BF). Our objectives were to study the time span during which women employed prior to pregnancy returned to work according to BF duration category, and to identify sociodemographic, behavioral and pregnancy characteristics of women who continued BF after returning to work.

**Methods:**

Information on BF mode and work status was prospectively collected in a French nation-wide birth cohort up to 1 year after delivery. Time of return to work according to BF category was addressed using Kaplan-Meier curves and Poisson regression adjusted on co-variates. Multiple logistic regression enabled to identify characteristics associated with the combination of BF with work.

**Results:**

Among 2480 women holding jobs prior to pregnancy, 82.0% returned to work within a year postpartum. Women who breastfed > 4 months returned at median of 6.5 months, whereas those who did not breastfeed at all returned to their jobs at 4.0 months, those who had breastfed for less than 1 month returned at 4.5 months, and those who had breastfed for 1 to 4 months returned at 4.0 months. Around one-third of women (34.5%) combined BF and work, and breastfed for a longer duration (median: 213 days, vs. 61 days for women who stopped BF before returning to work). Women born outside of France or who were self-employed were more likely to combine BF and work, while intermediate employees, manual workers, women who quitted smoking during pregnancy, who had smoked before and during pregnancy, or who had given birth by cesarean section were less likely to combine BF and work.

**Conclusion:**

Women who had breastfed for less than 4 months, or not at all, returned to their jobs at comparable times. This suggests that working women should be encouraged to breastfeed, even for a short duration. Moreover, only one-third of working women succeeded in combining BF and work, highlighting the need for a support system that would encourage flexibility.

## Background

Breastfeeding (BF) initiation has been shown to be insufficient, and mean BF duration falls under the recommended 6-month duration in most developed countries, despite its acknowledged benefits and public health actions [1]. Among several determinants, increasing attention has been paid to return to work (RTW) as a major constraint to initiate and continue BF [2–4]. Numerous previous studies consistently concluded that RTW before 12–16 weeks, compared with later or not at all, was associated with shorter BF duration [5–15]. Indeed, women themselves mentioned RTW as a reason for stopping BF [16–20], though not in all settings [21, 22], probably due to cultural norms and work support backgrounds. Moreover, women who planned to work after giving birth often did not begin BF, by anticipation. However, conclusions between studies are inconsistent [2, 7, 11–13, 23–27]. Methodological weaknesses, i.e. cross-sectional design or inaccurate data collection, limit our understanding of mechanisms involved in this phenomenon [28]. In addition, a potential bilateral relationship makes it difficult to interpret causality direction. Use of RTW as the outcome, and taking into account its confounders, could help to gain better insight into the complex relationship between BF behavior and RTW.

Although the relationship between RTW and BF cessation has been frequently examined, the combination of BF and work has rarely been studied in the general population [29, 30]. This type of behavior could be a strategy for encouraging BF and its duration. Solutions such as teleworking, flexible working hours and access to a room available for BF or for pumping milk would act as motivations for women to continue BF after RTW [30, 31]. Nevertheless, along with workplace conditions, characteristics of women who successfully combined BF and work have thus far been rarely described in Western countries.

Our objectives were to study the time span during which women employed prior to pregnancy returned to work according to BF duration category, and to identify sociodemographic, behavioral and pregnancy characteristics of women who continued BF after returning to work. This research was carried out in France, where most women have access to paid maternity leave, usually lasting around 12 weeks.

## Methods

### Population and follow-up

The inclusion process in the Epifane cohort, which was carried out between January and April 2012, has been described previously [32]. Two-stage random sampling was used. First, 136 maternity units were randomly selected proportionally to the yearly number of deliveries, stratified according to private/public status, equipment level of the maternity unit and five geographic areas. Second, after having checked for eligibility criteria, midwives included 25 mother-infant dyads 1 or 2 days after birth. Inclusion criteria were as follows: mothers aged 18 or over, not living in an institution, French-speaking or with access to help filling out questionnaires; gestational age at delivery ≥33 amenorrhea weeks; and no severe newborn pathology that required transfer to a specialized neonatology unit just after delivery. Follow-up was planned to last 12 months for each dyad. Mothers were interviewed by phone at 1, 4, 8 and 12 months. At the same time, they also filled in self-questionnaires using internet or regular mail.

### Measurements

Employment prior to pregnancy was reported at the maternity unit. At each further contact, women were asked whether they had returned to work and, if so, the exact date of return. In addition, their occupation was described 1 month after birth using a 10-category variable based on classification used by the French National Institute of Statistics and Economic Studies (https://www.insee.fr/fr/information/2400059) and grouped into 4 categories:

- “Farmers, artisans, merchants, etc.”, mainly including independent occupations (i.e. unsalaried);
- “Management”, including executive and managerial positions and other post-graduate occupations;
- “Intermediate employees”, i.e. those who attained an intermediate university level and work as teachers, health professionals (except for medical doctors, who were included in the “management” category), administrative employees, forewomen, etc.;
- “Manual workers”, grouping together occupations mainly involving factory production jobs.

BF status was assessed at the maternity unit and at each further follow-up appointment. Mothers were asked whether they were currently giving breastmilk, formula and all other liquids (and foods after 1 month). If they had been breastfeeding at the previous interview and had then begun to give formula or other liquids/foods, the infant’s age when they had begun was collected, as was the infant’s age (in months and weeks) when they stopped giving breastmilk or formula. Altogether, this information was used to define BF status over time using WHO definitions [33]: exclusive BF (no liquid other than breastmilk, except for vitamins and medication), formula or mixed BF (formula and breastmilk). “Any BF” (ABF) included infants who received breastmilk, exclusively or not, pumped or not. At 1-, 4- and 8-month interviews, women were questioned about the number of maternal milk feeds (including expressed milk) during the past few weeks, and about where the child usually slept at night.

Maternal characteristics were collected at birth (age, marital status, smoking before and during pregnancy, body weight and height before pregnancy) and at the 1-month interview (country of birth, education, parity). Information on birthweight (in grams), gestational age (in full amenorrhea weeks) and mode of delivery (vaginal/cesarean section) was collected by midwives in the medical records.

### Statistical analyses

Analyses are here limited to women employed before pregnancy, who gave birth to a singleton, and without missing data regarding BF mode at birth. Mode of feeding at birth was not known for 3 women. Dates of BF cessation (*n* = 102) and of RTW (*n* = 109) were imputed using medians of the interval between the last date of BF (or no work) and the first follow-up with changed status (no BF or RTW) (12 months for those lost to follow-up). In addition, RTW time was imputed using linear regression in 126 women who returned to work before the end of follow-up, but without a known interval. Other co-variates (age, education, occupation, marital status, birthplace, parity, body weight status before pregnancy, smoking status, birthweight, gestational age, and mode of delivery) were also imputed, thereby limiting selection bias due to non-random missing values. Sensitivity analyses were also performed using non-imputed variables for RTW time and ABF duration.

After computing initial probabilities of inclusion, a marginal calibration method was used to estimate final weights. Calibration involved percentages observed in the French National Perinatal Survey 2010 for age, marital status, education and type of pregnancy [34]. We used the “svyset” command (Stata® V.12) for taking into account the 2-stage sampling design and final weights in all analyses.

We first described the percentage of women returning to work during the first year after birth, and distribution of RTW time (25th, 50th and 75th percentiles) according to co-variate categories. Differences between categories were tested using the adjusted Wald test. To facilitate interpretation, we estimated RTW time according to the following ABF duration category, defined consistently with distribution observed in our sample: day 0 (no ABF), 1–28 days (short duration), 1–4 months (intermediate duration) and > 4 months (rather long duration). Kaplan-Meier curves of the probability of RTW over time according to these ABF categories were drawn. Since the hypothesis of risk proportionality was not met, Poisson regressions were used to estimate incidence rate ratios (IRR) of RTW time, in each ABF category, compared to no ABF. After univariate analyses, adjusted analysis included all co-variates for which time of RTW was variable, with a *p*-value < 0.20 in univariate analyses. As a complement, Poisson regression of the RTW time, including ABF duration as a continuous independent variable, was performed.

The second part of the analyses focused on women likely to combine ABF and work during the first year, i.e. women who breastfed at birth and returned to work within the first year. A combination of ABF and RTW was defined by date of ABF cessation later than the date of RTW (or no ABF cessation within the year of follow-up). In order to identify characteristics associated with a combination of ABF and work, we used logistic regression. For the dependent variable (combination of ABF and work), the comparison group included women who had stopped ABF before RTW. Selection of variables to be included in multivariate modelling was based on a *p*-value < 0.20 in univariate logistic regressions. Manual backward strategy was used to identify the final model that included statistically significant co-variates (*p* < 0.05). However, a co-variate with a *p*-value ≥0.05 might be kept if removing it modified the OR of other co-variates by over 10%.

## Results

A total of 3368 women were included in the Epifane cohort. Analyses regarding time of RTW were carried out in the 2480 women who worked prior to pregnancy. Characteristics associated with a combination of ABF and work were analyzed in the 1487 women who had worked before pregnancy and who returned to work within a year after delivery.

### Time of return to work in women who had worked prior to pregnancy

Characteristics of women and distribution of time at which they returned to work are presented in Table 1. A total of 82.0% of employed women returned to work within 1 year. Median time of RTW was 5.3 months (25th percentile: 3.1 months – 75th percentile: 8.8 months). The lowest percentages of RTW within 1 year were observed in women aged 35 or older, born outside of France, married, with elementary/middle school education, manual workers, obese women, smokers before and during pregnancy, or women having had 3 or more children (Table 1). The longest median times of RTW were observed in those same categories and in overweight women (Table 1). Percentages of RTW were comparable according to the mode of delivery (vaginal: 82.0%; cesarean section: 82.2%; *p* = 0.93), gestational age (≥37 weeks: 82.0%; 33–36 weeks: 81.7%; *p* = 0.95) and low birthweight (no: 81.9%; yes: 86.4%; *p* = 0.32).

**Table 1:** Characteristics of women who worked before pregnancy – Epifane Birth Cohort, 2012 (*n* = 2480)

	n	%^**a**^	Return to work			
			% before 1 y	Time (days)		
				25th	Median	75th
**Age**			***p*** **= 0.04**		***p*** **< 0.001**	
18–24 y	220	11.3	85.5	88	120	241
25–29 y	799	33.0	83.7	93	134	246
30–34 y	964	34.7	82.0	98	170	270
≥ 35 y	497	21.0	77.6	108	187	323
**Birthplace**			***p*** **< 0.001**		***p*** **= 0.01**	
Abroad	200	14.1	71.4	105	210	416
France	2280	85.9	83.8	95	152	249
**Matrimonial status**			***p*** **= 0.004**		***P*** **< 0.001**	
Married	1079	46.7	79.4	99	177	300
Unmarried	1401	53.3	84.3	93	141	248
**Education**			***p*** **< 0.001**		*p* = 0.054	
Elementary / Middle school	363	15.0	73.8	95	188	416
High school	580	23.1	76.7	95	168	334
University	1537	61.9	86.0	96	151	229
**Occupation before pregnancy**			***p*** **< 0.001**		***p*** **= 0.001**	
Farmers, artisans, merchants	74	2.8	85.2	56	96	170
Managers	546	22.4	88.9	97	147	214
Intermediate employees	1629	65.6	81.3	97	162	277
Manual workers	231	9.2	69.3	100	210	416
**Body weight status before pregnancy**			***p*** **= 0.001**		***p*** **= 0.004**	
Thin	178	7.3	82.2	97	165	282
Normal weight	1661	67.0	84.0	96	149	245
Overweight	433	17.0	79.4	98	189	305
Obese	208	8.7	71.9	91	186	416
**Smoking**			***p*** **= 0.001**		*p* = 0.07	
No	1691	69.6	82.5	96	161	264
Quit during pregnancy	425	16.5	86.0	93	134	239
Before & during pregnancy	364	13.9	74.9	100	180	416
**Parity**			***p*** **< 0.001**		***p*** **< 0.001**	
1	1115	46.0	90.2	90	118	210
2	1020	40.1	77.6	98	179	311
≥ 3	345	13.9	67.7	159	227	416

^a^Weighted percentages in each category

Characteristics statistically associated with BF duration categories were: age, birthplace, matrimonial status, education, occupation before pregnancy, smoking and parity (Additional Table 1). Body weight before pregnancy, mode of delivery, gestational age and birthweight were not associated with BF duration categories.

### Time of return to work according to breastfeeding duration

**Fig. 1:**
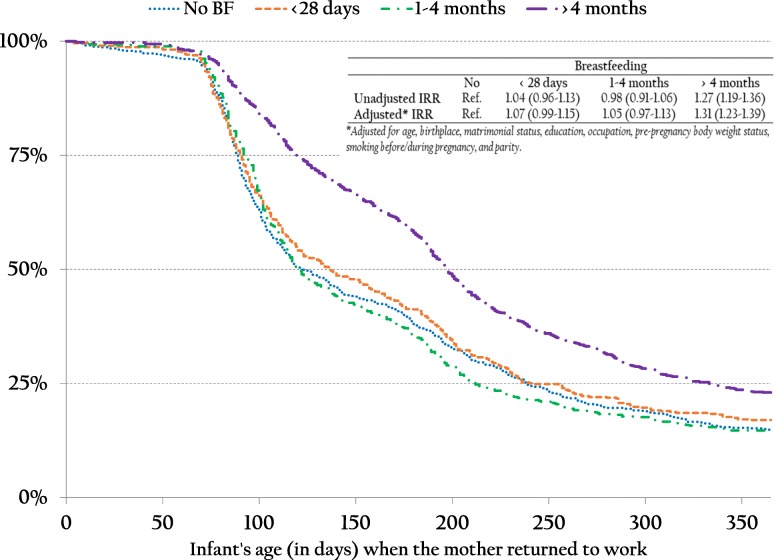
Probability of returning to work according to breastfeeding duration category (Kaplan-Meier curves and Poisson regression)

Since mean RTW time was statistically comparable between exclusive BF and mixed BF at birth (207 days (95%CI: 192–223) vs. 202 days (195–209)), subsequent analyses were based on ABF. Percentages of RTW within 1 year were 85.1% in women who did not breastfeed at all, 83.0% in women who breastfed at birth but for less than 1 month, 85.3% in women who breastfed for 1–4 months and 77.1% in those who breastfed for more than 4 months (*p* = 0.0002). Kaplan-Meier curves (Fig. 1) showed no difference in timing of RTW between the first three categories of ABF duration. Only women who breastfed for more than 4 months returned to work much later: at a median of 198 days (25th percentile: 120 days – 75th percentile: 333 days) versus 122 (89–239), 137 days (91–242) and 121 days (93–212) for the other categories, respectively.

After adjustment, Poisson regression showed that time of RTW was 31% later in women who had been breastfeeding for more than 4 months than for women who did not breastfeed at all (Table 2). Women falling within the shortest BF duration categories (< 1 month or 1–4 months) returned to work after a time period similar to that of women who did not breastfeed at all (aIRR: 1.07 [0.99–1.15] and 1.05 [0.97–1.13], respectively). Characteristics such as being born abroad (1.10 [1.01–1.20]), education lower than university level (high school: 1.10 [1.03–1.17]; elementary/middle school: 1.14 [1.06–1.24]), intermediate employment (1.10 [1.03–1.17]) or manual work (1.27 [1.15–1.41]), overweight (1.08 [1.02–1.15]) or obesity (1.13 [1.06–1.21]) before pregnancy, smoking before and during pregnancy (1.13 [1.06–1.21]) and multiparity (2 deliveries: 1.23 [1.16–1.30]; ≥ 3 deliveries: 1.48 [1.37–1.59]) remained statistically associated with later time of RTW (Table 2). When ABF duration instead of categories was included as a continuous variable in months, the adjusted IRR for time of RTW (in days) was 1.03 [1.02–1.04] (*p* < 0.0001).

**Table 2:** Association between women’s characteristics and time of return to work (Poisson regressions; *n* = 2480)

	Univariate models		Multivariate model	
	IRR	95%CI	IRR	95%CI
**Age**				
18–24 y	0.91	0.82–1.00	0.95	0.85–1.06
25–29 y	**0.93**	**0.88–0.99**	1.02	0.95–1.08
30–34 y	1		1	
≥ 35 y	**1.08**	**1.01–1.16**	1.02	0.95–1.09
**Birthplace**				
Abroad	**1.23**	**1.13–1.35**	**1.10**	**1.01–1.20**
France	1		1	
**Matrimonial status**				
Married	1		1	
Unmarried	**0.90**	**0.86–0.95**	0.97	0.92–1.02
**Education**				
Elementary / middle school	**1.21**	**1.18–1.30**	**1.14**	**1.06–1.24**
High school	**1.14**	**1.07–1.21**	**1.10**	**1.03–1.17**
University	1		1	
**Occupation before pregnancy**				
Farmers, artisans, merchants	0.83	0.67–1.04	**0.77**	**0.62–0.65**
Managers	1		1	
Intermediate employees	**1.13**	**1.06–1.20**	**1.10**	**1.03–1.17**
Manual workers	**1.35**	**1.23–1.49**	**1.27**	**1.15–1.41**
**Body weight status before pregnancy**				
Thin	1.05	0.95–1.16	1.04	0.95–1.15
Normal weight	1			
Overweight	**1.12**	**1.04–1.19**	**1.08**	**1.02–1.15**
Obese	**1.18**	**1.06–1.30**	**1.13**	**1.06–1.21**
**Smoking before pregnancy**				
No	1		1	
Quit during pregnancy	0.94	0.87–1.00	1.02	0.95–1.09
Before & during pregnancy	**1.11**	**1.03–1.19**	**1.13**	**1.06–1.21**
**Parity**				
1	1		1	
2	**1.26**	**1.19–1.34**	**1.23**	**1.16–1.30**
≥ 3	**1.58**	**1.48–1.69**	**1.48**	**1.37–1.59**
**BF durations**				
No BF	1		1	
< 1 month	1.04	0.96–1.13	1.07	0.99–1.15
1–4 months	0.98	0.91–1.06	1.05	0.97–1.13
> 4 months	**1.27**	**1.19–1.35**	**1.31**	**1.23–1.39**

*BF* breastfeeding, *CI* confidence interval, *IRR* incidence rate ratio

When analyses were performed using non-imputed variables for RTW and ABF, results were comparable. However, RTW time was significantly increased by 10% for ABF duration shorter than 1 month (aRRR: 1.10 [1.02–1.20]) (this aRRR was close to statistical significance when imputed variables were used: 1.07 [0.99–1.15] – Table 2).

### Characteristics associated with combination of breastfeeding and work

In women likely to combine ABF and work during the first year (*n* = 1487), 34.5% actually did so before the end of the first year. Most women who continued to breastfeed after RTW had exclusively breastfed at birth (93.7%), compared to 75.2% of those who ceased to breastfeed before RTW. Likewise, they significantly exclusively breastfed for a much longer duration (median number of days: 76 days vs. 10 days) or not (213 days vs. 61 days) (Table 3). At 1 and 4 months, the total number of feeds given was statistically higher than in women who ceased ABF before RTW (about one feed per day) (Table 3). At the same ages, infants of women who continued to breastfeed after RTW were significantly less likely to sleep in their own bedrooms, but rather in the parents’ bed or bedroom (Table 3).

**Table 3:** Breastfeeding and infant sleeping practices of women who continued to breastfeed after returning to work, compared with women who stopped breastfeeding before returning to work (*n* = 1487)

	ABF cessation before return to work		ABF continuation after return to work		p
	n		n		
**Exclusive BF at birth (%)**	993	75.2	494	93.7	< 0.001
**Median number of days**					
Exclusive BF	945	10	487	76	< 0.001
Any BF	993	61	494	213	< 0.001
**Mean (SE) number of feeds**					
***At 1 month***	528	7.0 (0.1)	473	8.2 (0.1)	< 0.001
***At 4 months***	152	4.6 (0.2)	405	5.3 (0.2)	0.005
During the day	145	3.9 (0.2)	399	4.2 (0.1)	0.20
During the night	145	0.7 (0.1)	399	1.1 (0.1)	< 0.001
**Baby’s sleeping place (%)**					
***At 1 month***	818		450		< 0.001
In his/her own room		34.8		22.2	
In parents’ room*		57.5		56.7	
In parents’ bed		7.5		20.2	
Other		0.3		0.9	
***At 4 months***	823		440		< 0.001
In his/her own room		63.9		50.9	
In parents’ room*		30.0		37.2	
In parents’ bed		1.1		6.6	
Other		4.9		5.4	

*But not in parents’ bed. *ABF* any breastfeeding, *BF* breastfeeding, *SE* standard error

According to final multivariate logistic regression **(**Table 4**)**, women born outside of France (aOR: 2.24 [1.49–3.36]) and farmwomen, artisans and merchants (versus managers: 2.23 [1.11–4.47]) were more likely to continue to breastfeed after RTW. Moreover, women who were intermediate employees (versus managers: 0.58 [0.44–0.76]) or manual workers (0.48 [0.27–0.88]), who had been smokers before pregnancy, whether quitting during pregnancy (versus non-smokers before pregnancy: 0.61 [0.44–0.84]) or not (0.49 [0.32–0.76]), and those who underwent cesarean section (0.69 [0.50–0.96]), were less likely to continue breastfeeding after RTW. When using non-imputed RTW time and ABF duration, the same ORs were estimated in this final model (data not shown).

**Table 4:** Association between women and pregnancy characteristics and continuing BF after return to work (logistic regressions; *n* = 1487)

	Univariate models		Final multivariate model	
	OR	95%CI	OR	95%CI
**Age**				
18–24 y	0.63	0.39–1.02		
25–29 y	**0.74**	**0.57–0.98**		
30–34 y	1			
≥ 35 y	1.01	0.74–1.39		
**Birthplace**				
Abroad	**2.28**	**1.55–3.36**	**2.24**	**1.49–3.36**
France	1		1	
**Matrimonial status**				
Married	1			
Unmarried	**0.71**	**0.57–0.90**		
**Education**				
Elementary / middle school	0.74	0.49–1.12	0.91	0.59–1.42
High school	**0.57**	**0.41–0.79**	0.74	0.52–1.04
University	1		1*	
**Occupation before pregnancy**				
Farmers, artisans, merchants	1.82	0.96–3.46	**2.23**	**1.11–4.47**
Managers	1		1	
Intermediate employees	**0.52**	**0.40–0.68**	**0.58**	**0.44–0.76**
Manual workers	**0.43**	**0.25–0.73**	**0.48**	**0.27–0.88**
**Body weight status before pregnancy**				
Thin	1.36	0.86–2.15		
Normal weight	1			
Overweight	1.05	0.77–1.42		
Obese	0.87	0.54–1.39		
**Smoking before pregnancy**				
No	1		1	
Quit during pregnancy	**0.57**	**0.41–0.78**	**0.61**	**0.44–0.84**
Before & during pregnancy	**0.38**	**0.25–0.57**	**0.49**	**0.32–0.76**
**Parity**				
1	1			
2	1.18	0.92–1.51		
≥ 3	1.23	0.85–1.78		
**Mode of delivery**				
Vaginal	1		1	
Cesarean	**0.72**	**0.52–0.98**	**0.69**	**0.50–0.96**
**Gestational age**				
≥ 37 weeks	1			
33–36 weeks	0.79	0.42–1.48		
**Birthweight**				
≥ 2500 g	1			
< 2500 g	0.54	0.27–1.05		

*Covariate kept in the final multivariate model, since removing it modified the OR of “Workers” by more than 10%. *CI* confidence interval, *OR* odds ratio

## Discussion

In our study carried out in France in 2012, eight out of ten employed women returned to work within a year, at a median of 5.3 months. We showed that no BF and ABF durations shorter than 4 months were associated with similar trends in RTW. Only women who breastfed for over 4 months returned to work at a later time. Furthermore, among breastfeeding women who returned to work within a year after delivery, one-third combined ABF and work. Birthplace, occupation, smoking status and mode of delivery were independently associated with combined ABF and work.

Comparison with other studies is limited due to variable durations of follow-up and different methods used to assess BF and work status. In our study, we used prospective repeated phone interviews for a one-year period to collect detailed information on infant feeding, which was the primary goal of the Epifane cohort [32]. In other studies using cross-sectional investigation, BF practice assessment was likely to lead to memory bias, especially when the interview was distant from the period in question. In contrast, we collected sparsely detailed information on work conditions after delivery. Other authors examined working status before pregnancy [21, 35], intention to return to work (not necessarily followed up) [9, 11, 23, 36], and various categories of time spans before RTW [6, 8–10, 15, 24]. In addition, groups of reference may have included mothers working before pregnancy, or not working at all, thereby affecting interpretation. In our study, we analyzed women who worked before pregnancy, enabling a more accurate interpretation. Moreover, since the RTW span is highly variable, it was considered here to be the outcome in survival analysis, thus enabling us to consider its potential determinants.

In our study, consistent with previous findings in other cohorts [8–10], ABF longer than 4 months was associated with a RTW 2 months later than for no ABF. It is difficult to determine whether women continued ABF because they were given the opportunity not to return to work before 4 months, or whether they chose to return to work later because they wished to breastfeed longer [37]. Nevertheless, Kaplan-Meier curves show no clear break at the end of the minimal legal maternity leave in France (12 weeks). In reality, paid maternity leave may vary under different circumstances, including legal provisions, i.e. shorter than 12 weeks for women who are self-employed, longer for women who have twins, a third pregnancy or major medical complications, women who postpone part of the prenatal leave after birth or who take annual leave, or have other favorable conditions agreed upon with their employer. The French situation is not transposable to all settings, which depend on the legal environment [2]. In-depth interviews are needed to better understand how work constraints influence the individual decision to breastfeed.

Another insightful finding was RTW time statistically comparable between women who did not breastfeed at all and those who breastfed for less than 4 months. Therefore, we hypothesize that BF initiation may not necessarily be dependent on a planned RTW [2, 36]. Determinants of BF initiation other than planned RTW should therefore be taken into account. For instance, pregnancy complications are considered to be strong determinants of BF initiation [4, 38], but they did not affect the RTW time in our study. “Farmwomen, artisans and merchants” were likely to return to work much earlier, but they were also more prone to breastfeeding for a longer duration. Flexibility in working hours therefore appears to be a key determinant in BF initiation and continuation [30]. In addition to occupation, full-time or part-time work status may also be a decisive factor, as shown in previous studies [39, 40]. Such information was not available here, and our goal was to identify general characteristics of women who combined BF and work.

Indeed, women who combined BF and work showed significant specificities. First, exclusive BF since birth and on demand (loosely based on the number of feeds and on where the child slept) (Table 3) might augment the possibility of continuing BF after RTW. Second, self-employed women who may have some flexibility (“Farmers, artisans and merchants”) were most likely to continue BF. In contrast, manual workers and “intermediate employees” (i.e. with subordinate positions) were less likely to be able to continue ABF [41]. In an intermediate situation, and despite early RTW and other working constraints, managers may have continued ABF because it was compatible with their work organization and also because of the health benefits they expected for their child. The fact that, in our study, women who continued to breastfeed after RTW were more likely to be non-smokers before pregnancy also underlines a potentially higher degree of health awareness.

Though they were less likely to return to work within the child’s first year, women born outside of France were more likely to combine ABF and work, regardless of other co-variates. Birthplace abroad was previously highlighted as a prominent BF determinant in developed countries [42]: community norms and dissemination across generations may account for such findings. Thus, ABF behavior of foreign women who return to work would appear to be influenced by such conditions. The manner in which they succeed in combining ABF and work could help in developing actions destined for native-born mothers.

The primary objective of the Epifane cohort was to describe infant feeding during the first year of life. We therefore collected extremely detailed information [32]. However, information related to maternal working conditions is more limited, as underlined above. Moreover, though a large set of co-variates was taken into account, we cannot rule out residual confounding in the relationship between BF duration and RTW time, such as breastfeeding intentions, encouragement for breastfeeding (from the family, etc.), and income, which were not collected in this cohort. In addition, due to the short duration of exclusive BF in France, its specific analysis was not carried out, although this represents a target to be attained. Finally, despite strict instructions given to interviewers not to influence mothers, repeated questions on BF may have modified related behavior and yielded an overestimated percentage of women combining ABF and work. Finally, our results require confirmation by further studies in other settings.

## Conclusion

Our findings may have important consequences for BF promotion. In contrast to generally held ideas, RTW does not necessarily prevent BF initiation or continuation, although this phenomenon is measurable only for BF duration of less than 4 months. Moreover, only one-third of women succeeded in combining work and BF. Indeed, working conditions, as described in our study via occupational categories, would appear to be of crucial importance. Finally, much improvement is needed in research, and at the public health level, so as to include widespread dissemination of information directed toward women who wish to continue breastfeeding after returning to work.

## Supplementary information

**Additional file 1: Table S1.** Any breastfeeding (ABF) duration category according to characteristics of women who worked before pregnancy, Epifane Birth Cohort, 2012 (*n* = 2480).

## Data Availability

The datasets used and/or analyzed during the current study are available from the corresponding author on reasonable request.

## Abbreviations

ABF: Any breastfeeding
BF: Breastfeeding
CI: Confidence interval
IRR: Incidence rate ratios
OR: Odds ratio
RTW: Return to work
SE: Standard error
