# Supplementary material for: Combining breastfeeding and work: findings from the Epifane population-based birth cohort
Source: BMC Pregnancy Childbirth. 2020 Feb 17;20:110. doi: 10.1186/s12884-020-2801-x (PMC7027215; doi:10.1186/s12884-020-2801-x)
Supplement: Supplementary file 1 — Additional file 1: Table S1. Any breastfeeding (ABF) duration category according to characteristics of women who worked before pregnancy, Epifane Birth Cohort, 2012 (n = 2480). [file 12884_2020_2801_MOESM1_ESM.docx]

**Additional Table 1: Any breastfeeding duration category according to characteristics of women who worked prior to pregnancy – Epifane Birth Cohort, 2012 (n=2,480)**

|  | **ABF duration category** | | | |  |
| --- | --- | --- | --- | --- | --- |
|  | **No ABF** | **< 1 Mo** | **1-4 Mo** | **> 4 Mo** | **p-value*** |
| **Age** |  |  |  |  | **<0.0001** |
| 18-24 y | 32.7 | 26.2 | 18.4 | 22.6 |  |
| 25-29 y | 25.7 | 19.5 | 26.5 | 28.2 |  |
| 30-34 y | 23.7 | 16.6 | 20.9 | 38.8 |  |
| ≥ 35 y | 25.2 | 14.0 | 18.1 | 42.6 |  |
| **Birthplace** |  |  |  |  | **<0.0001** |
| Abroad | 10.9 | 12.3 | 16.2 | 60.5 |  |
| France | 28.1 | 19.1 | 22.8 | 30.0 |  |
| **Matrimonial status** |  |  |  |  | **<0.0001** |
| Married | 22.3 | 15.6 | 20.7 | 41.4 |  |
| Unmarried | 28.6 | 20.4 | 22.9 | 28.1 |  |
| **Education** |  |  |  |  | **<0.0001** |
| Elementary / Middle school | 35.6 | 26.4 | 14.5 | 23.5 |  |
| High school | 32.0 | 19.8 | 22.2 | 26.0 |  |
| University | 20.9 | 15.5 | 23.6 | 40.0 |  |
| **Occupation before pregnancy** | |  |  |  | **<0.0001** |
| Farmers, artisans, merchants | 25.8 | 14.4 | 16.3 | 43.5 |  |
| Managers | 16.7 | 12.3 | 25.2 | 45.7 |  |
| Intermediate employees | 27.6 | 19.6 | 21.3 | 31.5 |  |
| Manual workers | 33.8 | 23.1 | 19.7 | 23.4 |  |
| **Body weight status before pregnancy** | |  |  |  | 0.26 |
| Thin | 28.5 | 19.6 | 15.7 | 36.2 |  |
| Normal weight | 24.9 | 17.4 | 22.6 | 35.1 |  |
| Overweight | 25.1 | 21.5 | 20.9 | 32.5 |  |
| Obese | 30.6 | 15.8 | 23.1 | 30.5 |  |
| **Smoking** |  |  |  |  | **<0.0001** |
| No | 22.9 | 15.9 | 22.1 | 39.1 |  |
| Quit during pregnancy | 27.4 | 24.4 | 23.2 | 25.0 |  |
| Before & during pregnancy | 37.5 | 21.9 | 19.1 | 21.5 |  |
| **Parity** |  |  |  |  | **<0.0001** |
| 1 | 24.1 | 20.7 | 25.2 | 30.0 |  |
| 2 | 27.6 | 16.6 | 19.6 | 36.2 |  |
| ≥ 3 | 25.2 | 14.1 | 17.6 | 43.0 |  |
| **Mode of delivery** |  |  |  |  | 0.12 |
| Vaginal | 24.7 | 18.3 | 21.9 | 35.1 |  |
| Cesarean | 30.2 | 17.4 | 21.7 | 30.7 |  |
| **Gestational age** |  |  |  |  | 0.81 |
| ≥ 37 weeks | 25.7 | 18.1 | 21.7 | 34.4 |  |
| 33-36 weeks | 24.4 | 17.6 | 26.3 | 31.7 |  |
| **Birthweight** |  |  |  |  | 0.26 |
| ≥ 2,500 g | 25.9 | 18.2 | 21.6 | 34.3 |  |
| < 2,500 g | 19.4 | 17.0 | 30.8 | 32.8 |  |

*Weighted percentages. *Comparison between the four categories (Design-based Pearson Chi Square with the Rao and Scott correction). ABF: any breastfeeding.*
